# Antimicrobial and Cytotoxicity Activities of Medicinal Plants against *Salmonella gallinarum* Isolated from Chickens

**DOI:** 10.1155/2022/2294120

**Published:** 2022-02-28

**Authors:** Mwanaisha Mkangara, Fulgence N. Mpenda

**Affiliations:** ^1^Department of Science and Laboratory Technology, Dar es Salaam Institute of Technology, P. O. Box 2958, Dar es Salaam, Tanzania; ^2^Department of Molecular Biology and Biotechnology, University of Dar es Salaam, P. O. Box 35179, Dar es Salaam, Tanzania

## Abstract

Medicinal plants have been the good source of treatment for different ailments of humans as well as animals for centuries. However, in Tanzania, few plants were investigated for their efficacy against various diseases of chickens. In the present study, four medicinal plants were investigated against *Salmonella gallinarum* isolated from chickens. The minimum inhibitory concentration (MIC) using the broth microdilution methods and minimum bactericidal concentration (MBCs) were used to evaluate the activities of plants against chicken salmonellosis. For the safety of chickens against the toxicity of plants, the cytotoxicity assay was determined using a brine shrimp lethality test. *Aloe secundiflora* leaf ethyl acetate (ALEA), *Aloe rabaiensis* leaf methanolic (ArM), *Aloe rabaiensis* leaf ethyl acetate (ArLEA), and *Punica granatum* leaf ethyl acetate (PGLEA) extracts exhibited the highest MIC (0.3906 mg/mL) and MBC (3.125 mg/mL), respectively. The *Dolichos kilimandscharicus* tuber ethyl acetate (DTEA) and *Dolichos kilimandscharicus* tuber pet ether (DTPE) extracts displayed MIC of 1.563 mg/mL and 12.50 mg/mL and MBC of 12.50 mg/mL and 25.50 mg/mL, respectively. The highest LC_50_ values exhibited in *Dolichos kilimandscharicus* ranged from 7.937 × 10^−4^ mg/mL to 7.242 × 10^−2^ mg/mL for pet ether and methanolic extracts, respectively, while ALEA extract exhibited LC_50_ of 7.645 × 10^−3^ mg/mL. Generally, the extracts with MIC 0.3906 mg/mL and MBC 3.125 mg/mL demonstrated the highest antibacterial activity with low toxicity efficient to manage chicken salmonellosis. *Dolichos kilimandscharicus*, which exhibited higher toxicity, warrants further investigation on insecticidal and anticancer agents.

## 1. Introduction

*Salmonella* spp. is a Gram-negative facultative anaerobic bacterium that belongs to the family *Enterobacteriaceae* [1]. The bacterium is zoonotic, affecting both chickens and humans [2]. The management of chickens with salmonellosis makes human the most affected individual than any other species [3]. However, salmonellosis in humans can also occur through eating contaminated food of animal origin including cattle, pork, and other poultry species [4, 5]. Globally, there are 94 million cases of gastroenteritis associated with salmonellosis, which account for about 155,000 deaths each year [6–8]. The poultry sector alone accounts for up to 50% of salmonellosis outbreaks in humans [9]. Therefore, investigating for proper and affordable medication against salmonellosis in chickens is vital for the development of the chicken industry as well as the improvement of public health.

The increasing frequency of antibiotic resistance strains from bacterial, virus, fungi, and protozoa and failure of several drugs developed recently have shifted the global interest to plant-based products [10]. The fact is that medicinal plants are the primary source of bioactive compounds potential for the development of nutritional and pharmaceutical drugs [11, 12]. Medicinal plants, unlike most antibiotics with a single target site, react with pathogens in multiple ways [13]. Different from antibiotics, bioactive compounds from medicinal plants can simultaneously disrupt the cellular membrane of a pathogen, stimulate the immune system of the host, protect intestinal mucosa from pathogen colonization, and promote the growth of beneficial bacteria [14]. For example, the bark of cinnamon (*Cinnamomum zeylanicum*) significantly reduced *Salmonella enterica* in cecal contents of infected chicken by disrupting bacterial cell membrane without affecting the total cecal endogenous population [15–17]. This observation demonstrates how bioactive compounds from medicinal plants promote beneficial bacteria, which later outcompete pathogens in resources and turn to improve the immune system of the host against diseases [18].

In Tanzania, chicken farming is constantly growing and contributes to 16% of the livestock GDP, 3% of agricultural sector GDP, and 1% of national GDP [19]. However, infectious diseases, insufficient veterinary service, and unaccommodated prices of effective drugs mostly to the smallholder farmers are among the setbacks of chicken farming in the country [20].

Therefore, the study investigated the antibacterial and cytotoxicity activities of four Tanzanian medicinal plants, namely, *Aloe secundiflora* var sabolifera, *Aloe rabaiensis*, *Punica granatum,* and *Dolichos kilimandscharicus* against *Salmonella gallinarum* isolated from village chickens.

## 2. Materials and Methods

### 2.1. Plant Materials Collection

The plant materials were collected from August to September 2020. The leaf of *Aloe secundiflora* var sabolifera was obtained from Makuyuni, Arusha (3.1919 S, 36.5518 E, at altitude 1090 M), *Aloe rabaiensis* was obtained around Lake Jipe in Mwanga, Kilimanjaro (3.34882 S, 37.44202 E at altitude 718 M), *Punica granatum* leaf, fruit peel, and seed were obtained from Ngurdoto, Arusha (3.1919S, 36.5518 E, at altitude 1332 M), and *Dolichos kilimandscharicus* tuber was obtained from Moshi, Kilimanjaro (3.21 S, 37.2 E, at altitude 1220 M). Collected plants were identified by a Botanist from Tanzania Pesticide Research Institute (TPRI) and the voucher specimen number; ARH 403, PGH 507, DKH 212, and ASH 325 for *A. rabaiensis*, *P. granatum*, *D. kilimandscharicus,* and *A. secundiflora*, respectively, were deposited in the herbarium at TPRI.

### 2.2. Plant Materials Processing

Plant materials were washed with running tap water followed by distilled water to remove dust and soil. After washing, the leaves of *A. secundiflora* var sabolifera and *A. rabaiensis*, the tuber of *D. kilimandscharicus,* fruit peel, and leaves of *P. granatum* were chopped into small pieces and then air-dried separately under the shade for three weeks. The seeds of *P. granatum* were also air-dried under the shade separately without washing them. After drying the plant, materials were pulverized by a mill machine (Swinging Traditional Chinese Machine Pulverizer Diaxiang electronic equipment, DXF- 20D, China) into fine particles and kept in food bags and stored at room temperature (25°C) for two weeks until utilization.

### 2.3. Chemical and Reagents

Nutrient agar and broth, Selenite F broth, Rappaport Vassiliadis broth, SIM media, Tryptone Soya Broth, MacConkey, Triple Sugar Iron (TSI), and Xylose Lysine Deoxycholate (XLD) agar and Buffered peptone water were sourced from Hi-Media Laboratories Pvt Ltd (Mumbai-India). Dimethyl sulfoxide (DMSO) was purchased from Sigma® (Poole, Dorset, UK). Analytical solvents were procured from RFCL Limited (Haryana-India). Brine Shrimp eggs were purchased from Aquaculture innovations (Grahamstown 6140, South Africa), and finally, Gentadox was purchased from Interchemie werken, Holland.

### 2.4. Extraction Procedure

The sequential extraction was done using solvents in order of increasing polarity. One hundred and twenty-five grams (125 g) of each pulverized plant material was socked in extracting solvent (1000 ml, 72 h) in a shaker (Dragon lab, USA). The solvent used included pet ether, ethyl acetate, and methanol. The socked plant materials were then filtered using cotton wool and Whatman number 1 filter paper. The filtrates were concentrated using a Rotary evaporator (Heidolph, Germany), and later methanolic extract was further evaporated in a water bath at 40°C for 24 h. The extracts obtained were weighed and kept in the refrigerator at 4°C for further use.

### 2.5. Faecal Samples Collection

A total of 360 village chickens aged 3 to 5 months were used in the study. The chickens were randomly picked from farmers in two wards, upland and lowland Tengeru-Arusha, Tanzania, from September 2020 to November 2020. An average of five chickens per household was used to collect 10 g of fecal samples from the cloaca of each chicken. The collection of fecal samples followed the diarrhea outbreak in adult chickens with white-yellowish coloration from the vent. The fecal sample was kept in a universal bottle containing 25 mL of buffered peptone water and incubated at 35°C for 18 h.

### 2.6. Isolation of Salmonella spp. from Faecal Samples

The 1 mL of incubated fecal samples kept in buffered peptone water was enriched in 9 mL of Tryptone Soya Broth (TSB) and incubated at 37°C for 24 h. Later, 0.5 mL of enriched broth was transferred in 9.5 mL of selective broth (Rappaport Vassiliadis broth) and incubated at 42°C for 48 h. Consequently, the sterile cotton swab dipped in Rappaport Vassiliadis broth was streaked on MacConkey agar and Xylose Lysine Deoxycholate (XLD) agar and then incubated at 37°C for 24–36 h for a single pure colony. The colonies from MacConkey agar appeared colorless and translucent, while colonies from XLD agar appeared red with a black center after prolonged incubation.

### 2.7. Identification of Salmonella spp. by Biochemical Reaction

Biochemical identification of *Salmonella* species was made as described by Office International des Epizooties (OIE) Manual of diagnostic tests and vaccines for terrestrial animals, volume 1, 2008 [21].

### 2.8. Evaluation of Antibacterial Activity

Minimum inhibitory concentrations (MICs) were used to determine antimicrobial activity by microdilution methods using 96-well microtiter plates as per Eloff [22] with minor modifications. The plates were first preloaded with 50 *μ*L of the nutrient broth in each well followed by the addition of 50 *μ*L of the extract (100 mg/mL prepared in 5% DMSO) into the first wells of each row to make a total volume of 100 *μ*L in the first wells. After thorough mixing, 50 *μ*L was drawn from each of the first row wells and put into the subsequent rows to the last wells, where the drawn 50 *μ*L was discarded. Thereafter, 50 *μ*L of *Salmonella* suspension (0.5Mac Farland standard turbidity, a suspension containing about 1.5 × 10^8^ cfu mL^−1^) was then added to each well to make the final volume of 100 *μ*L. The rows containing 0.05 mg/mL of Gentadox (50 *μ*L) were positive control used as a standard drug for chicken salmonellosis, and the wells, which contained DMSO (5%), nutrient broth, and bacteria in triplicate form, were used as negative control. The plates were then incubated at 37°C for 24 h. For each extract, MICs were determined by adding 10 *μ*L of 0.02% p-iodonitrotetrazolium (INT) chloride dye in each well, followed by incubation for 1 h at 32°C. A color change indicated bacterial growth. Colour changed to pink is an indicator of the active growth of bacteria. The lowest concentration of extract, which showed no bacterial growth, was considered as MIC.

Minimum Bactericidal Concentration (MBC) was obtained by subculturing the contents from 96-well microtiter plates, where MIC value was read including all wells above the MIC. The wells, which showed no visible growth of *Salmonella* on McConkey agar, were considered as MBC. According to Qi et al. [23], the MBC is the lowest concentration of antibacterial agent required to kill more than 99.9% of the initial bacterial population, where no visible growth of the bacteria is observed on the agar plates.

### 2.9. Brine Shrimp Lethality Test

Cytotoxicity of the extracts was evaluated using brine shrimp of *Artemia Salina* Leach according to Meyer et al. [24], with minor modification. The medicinal plant extracts from *A. secundiflora* var sabolifera, *A. rabaiensis*, *D. kilimandscharicus,* and *P. granatum* were used in this study. The stock solution (40 mg/mL) was made by dissolving extracts to 5% DMSO to make different levels of concentration (240, 120, 80, 40, 24, and 8 *µ*g/mL) [25]. The levels of concentration were made from different volumes of a stock solution, which were then added in vials and adjusted to 5 mL of artificial seawater (3.8 g/L). Each level of concentration was tested in duplicate. The negative control contained artificial seawater, brine shrimps, and DMSO (5%) only. The positive control contained cyclophosphamides (50 mg/kg) prepared by dissolving 40 mg/mL similar to the stock solution. The light was used to incubate the vials for 48 h with a constant Oxygen gas supply. After this period, the dead larvae (dead nauplii) were counted, and the means mortality was subjected to analysis using Fig. P computer program (Biosoft Inc, USA).

### 2.10. Data Analysis

Mean results of brine shrimp mortality against logarithms of concentration were plotted using figure P computer program (Biosoft Inc., USA). Fig. P computer program gives regression equations, which were used to calculate LC_16_, LC_50_, LC_84_ values. The confidence intervals (95% CI) were calculated according to Litchfield and Wilcoxon [26]. The LC_50_ value higher than 100 *µ*g/mL equivalent to 1 × 10^−1^ mg/mL was considered nontoxic and below it was considered toxic [27].

## 3. Results and Discussion

Antibacterial activity of plant extracts from *A. secundiflora* Engl var sabolifera*, A. rabaiensis* Rendle, *D. kilimandscharicus Taub*, and *P. granatum* Lin was evaluated against *Salmonella gallinarum* isolated from village chickens. Cytotoxicity activities of the above-named plant extracts were also evaluated against brine shrimps. The results of MIC and MBC of *A. secundiflora* leaf ethyl acetate, *A. rabaiensis* leaf ethyl acetate, *A. rabaiensis* leaf methanolic, *P. granatum* seed ethyl acetate, and *P. granatum* leaf ethyl acetate extracts revealed good antibacterial agents. The extracts showed higher activity and low toxicity as summarized in Tables 1 and 2. The details of biochemical reactions of isolated *Salmonella* spp., antibacterial, and cytotoxicity activities of named plants are explained hereunder.

### 3.1. Biochemical Reactions of Salmonella spp

**Figure 1:**
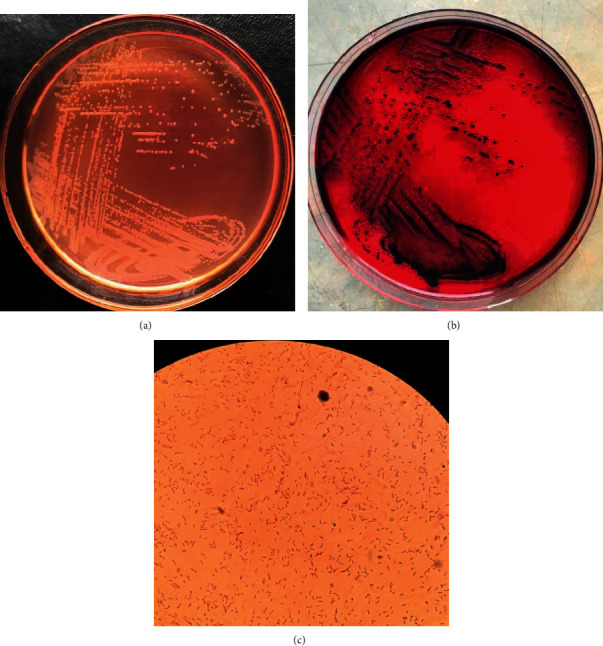
(a): *Salmonella gallinarum* colonies in MacConkey agar. (b) *Salmonella gallinarum* colonies in xylose lysine deoxycholate (XLD) agar. (c) Rod shaped of *Salmonella gallinarum* (×100) after Gram staining.

Biochemical identification systems of bacteria are based on one or a combination of factors such as the utilization of carbon source, change in pH (carbohydrate is utilized pH acidic, nitrogen is released pH alkaline), or detection of growth of the organism [28]. Out of 360 samples of bacteria isolated from fecal samples of chickens and plated on two selective solid agars, 162 (45%) isolates were preliminarily thought to be *Salmonella* species after morphological observation of colonies. The colonies were whitish, transparent, shining, and convex shape on MacConkey agar (Figure 1(a)). Similar colonies on xylose lysine deoxycholate (XLD) agar were transparent red with black centers (Figure 1(b)). Microscopically, the colons were pink rods after Gram staining (Figure 1(c)). According to Ranjbar et al. [29], XLD agar is among the sought media for the growth of *Salmonella* spp. The ingredients in XLD agar include sodium deoxycholate and an indicator system of phenol red combined with sugars (xylose, sucrose, and lactose), sodium thiosulphate, and iron all together with a slightly alkaline pH of 7.4 [30]. The carbohydrate catabolism of xylose during the growth of *Salmonella* lowers the pH from slightly alkaline to acidic, and the colonies grow with red coloration [31]. The black centers in colonies of *Salmonella* spp. are the result of an evolution of hydrogen sulfide from thiosulphate.

The confirmation of *S. gallinarum* was done by biochemical reactions. The red colon with a black center on XLD agar was then subcultured on nutrient agar. The pure colon from nutrient agar undergoes Gram staining, enzymes and sugar utilization test, hydrogen sulfide evolution, and motility tests. The biochemical tests identified 138 (38.3%) isolates as *Salmonella gallinarum* (Table 3). Out of 138 (38.3%) isolates of *S. gallinarum*, a total of 60 (16.7%) isolates were the cultures of fecal samples from upland Tengeru chickens and 78 (21.6%) from lowland Tengeru chickens (Table 3). In reference to these findings, which require further investigations, probably the river flowing from highland Tengeru to lowland Tengeru might also be the other source of spread of *Salmonella* as the chickens were found scavenging around the river and use the river water as a source of drinking water.

Nonmotility test observed in *Salmonella* samples identified from this study grouped the bacteria into either *Salmonella gallinarum* or *Salmonella pullorum*. The fact is that nonmotile *Salmonella* species is a characteristic of only *S. gallinarum* and *S. pullorum* in all subspecies of *Salmonella enterica* [32]. The sugar utilization tests on *S. gallinarum* revealed ferment maltose, dulcitol, fructose, and dextrose and not sucrose or lactose in sugar utilization (Table 4). These findings corroborate Sannat et al. [33] who identified *S. gallinarum* after fermenting dulcitol, maltose, and glucose, and *S. pullorum* after fermenting rhamnose and glucose with gas butt not dulcitol and maltose. Therefore, the sugar utilization, Gram staining, enzymes reactions, and motility tests of the biochemically tested *Salmonella* spp. demonstrated the presence of *S. gallinarum* in 138 (38.3%) samples of bacteria collected from 360 fecal samples of chickens in two wards (Tables 3 and 4).

### 3.2. Determination of Antibacterial Activity

Antibacterial activity of pet ether and ethyl acetate and methanolic extracts of *A. secundiflora* (leaf), *A. rabaiensis* (leaf), *P. granatum* (leaf, seed, and fruit peel), and *D. kilimandscharicus* (tuber) were evaluated against *S. gallinarum* isolated from chickens. Out of 17 extracts, 5 (29.41%), namely, *P. granatum* seed and leaf ethyl acetate, *A. secundiflora* leaf ethyl acetate, *A. rabaiensis* leaf methanolic, and *A. rabaiensis* leaf ethyl acetate extracts, indicated the highest antibacterial activity against *S. gallinarum*, with MIC value of 0.309 mg/mL. However, 12 extracts (70.59%) represented moderate antibacterial activity with their MIC ranging from 0.781 mg/mL to 12.5 mg/mL. In comparison to the MIC, the MBCs of all plant extracts tested are two or three times their MICs (Table 1). The antibacterial activity illustrated from this study is supported by other scholars such as Kaingu et al. [34] who investigated the anticoccidial effects of *Aloe secundiflora* against *Eimeria tenella* in broiler chickens after preventing death and severity of bloody diarrhea. Others are Msoffe and Mbilu [35] who investigated *A. secundiflora* against the *Candida albicans* with inhibition zones of 11.46 ± 0.69 to 16.66 ± 1.09 mm at the concentration of 20 to 100 *µ*L, respectively. According to Waihenya et al. [36], the severity and mortality of chickens infected with Newcastle disease virus were significantly reduced after treating with *Aloe secundiflora*. On the other hand, Mariita et al. [37] studied the antitubercular of *A. secundiflora* and observed the lowest MIC of 0.5 mg/mL, which inhibited 99% of *Mycobacterium tuberculosis*. The antibacterial activity of *A. secundiflora* was also revealed in *P. aeruginosa*, *E. coli*, *S. aureus,* and *S. typhi* with inhibition zones ≥9.00 mm, and MIC value ranged from 3 to 11 mg/mL [8, 38]. The observable antimicrobial activities of *Aloe secundiflora* are attributed to a mixture of phenolic compounds mainly anthrones, chromones, and phenylpyrones, and their derivatives [39, 40]. Similar to these findings, phytoconstituents in *Aloe secundiflora* revealed the presence of flavonoid catechins, which is capable of inhibiting the actions of DNA polymerase in bacteria and bind and damage the bacterial cell membrane, which finally increases permeability and leads to cell lysis [41, 42].

Historically, *Punica granatum* is known for its therapeutic activity to ameliorate diseases since Roman times [43, 44]. In traditional medicine, pomegranate leaves, flowers, roots, and fruit have been used to treat microbial infections, diarrhea, helminthiasis, dysentery, haemorrhage, acidosis, and respiratory and cardiac diseases [45, 46]. In the present study, *P. granatum* showed the highest antibacterial activity against *S. gallinarum* with MIC values ranging from 04500.390 mg/mL to 6.25 mg/mL (Table 1). A study by Haidari et al. [47] investigated *P. granatum* against the human influenza A virus, and the findings were promising due to polyphenols from pomegranate with active ingredient punicalagin, ellagic acid, and hydrolyzable tannins. The study by Abou El-Nour [48] reported the antibacterial activity of pomegranate peel extract against *S. typhi*, *E. coli,* and *S. aureus* with inhibition zone ranging from 8 to 15 mm. Similar findings by Duman et al. [49] reported antibacterial efficacy of *P. granatum* fruit peel extract against *V. cholera*, *S. typhi, S. flexneri, S. dysenteriae, E. coli,* and *S. aureus* with zones of inhibition ranging from 12 to 31 mm. The observable activities in *P. granatum* are associated with punicalagin, ellagic acid, ellagitannins, and gallotannins, which are essential constituents in *Pomegranate* with antibacterial, antiviral, and antifungal properties [50–52].

Root and tuber of *D. kilimandscharicus* are known for antifungal and ant-molluscicidal properties attributed by saponins [53, 54]. In this study, *D. kilimandscharicus* tuber extracts revealed the highest toxicity with LC_50_ values of 7.937 × 10^−4^ mg/mL, 3.5192 × 10^−3^ mg/mL, 1.468 × 10^−2^ mg/mL, and 7.242 × 10^−2^ mg/mL for pet ether, water, ethyl acetate, and methanolic extracts, respectively (Table 2). The results corroborated Sithole [55] who observed the efficacy of saponins as a natural detergent in *D. Kilimandscharicus* with antimicrobial and cholesterol-lowering anticancer compounds.

*A. rabaiensis* leaf methanolic and *A. rabaiensis* leaf ethyl acetate extracts inhibited the highest activity against *S. gallinarum* in *in-vitro* assay with MIC value of 0.390 mg/mL, and MBC ranged from 1.563 to 3.125 mg/mL (Table 1). The activity is due to phenolic constituents chromone, anthraquinone, and anthrone with antibacterial properties [56, 57].

According to Mushi et al. [58], MIC values are interpreted as follows: 0.05–0.5 mg/mL is strong activity, 0.6–1.5 mg/mL is moderate activity, and ˃1.5 mg/mL is weak activity. The tested plants in this study have inhibited *S. gallinarum* in *in vitro* assays; thus, the study suggests further investigation of plants in the experimenting chickens to assess their effectiveness in *in vivo* assays.

### 3.3. Brine Shrimp Lethality Test

Cytotoxicity efficacies of plant extracts evaluated in this study are shown in Table 2. Generally, pet ether, water, and ethyl acetate extracts of *D. kilimandscharicus* were observed to have the highest toxicity against brine shrimp larva with LC_50_ value of 7.937 × 10^−4^ mg/mL, 3.519 × 10^−3^ mg/mL, and 1.468 × 10^−2^ mg/mL, respectively. *Punica granatum* seed ethyl acetate and *A. Secundiflora* leaf ethyl acetate exhibited toxicity with LC_50_ values of 6.428 × 10^−3^ mg/mL and 7.645 × 10^−3^ mg/mL, respectively. However, their antibacterial activities are moderate. Therefore, the candidates qualify to be antitumor, anticancer, or insecticidal agents. According to Moshi et al. [27], the toxicity of plant extracts is termed nontoxic when LC_50_ > 1 × 10^−1^ mg/mL, and vice versa. *Punica granatum* fruit peel methanol, *P. granatum* leaf ethyl acetate, *A. rabaiensis* leaf water, *A. secundiflora* leaf methanol, *P. granatum* leaf pet ether, *P. granatum* seed methanol, and *P. granatum* fruit peel ethyl acetate extracts have LC_50_ values of 1.245 mg/ml, 1.245 mg/mL, 5.016 × 10^−1^ mg/mL, 3.902 × 10^−1^ mg/mL, 2.302 × 10^−1^ mg/mL, 2.082 × 10^−1^ mg/mL, 1.720 × 10^−1^ mg/mL, and 1.512 × 10^−1^ mg/mL, respectively (Table 2). These extracts are nontoxic and have shown higher antimicrobial efficacy against *Salmonella* species isolated from chickens.

## 4. Conclusion

The use of medicinal plants with varieties of secondary metabolites that react against pathogenic microbes in different ways to antibiotics is necessary for the management of *S. gallinarum*. The plants investigated in this study are worthwhile bacteriostatic as well as bactericidal in the management of bacterial infections including salmonellosis. The *A. rabaiensis*, *A. secundiflora,* and *P. granatum* revealed appreciable ranges of MIC and MBC as candidates with antibacterial properties. The *D. kilimandscharicus* extracts revealed low antibacterial activity against *S. gallinarum*. However, their cytotoxicity efficacies are highest compared to other plants. Therefore, *D. kilimandscharicus* warrants further investigation against tumors and insects.

## Figures and Tables

**Table 1:** MIC and MBC of plant extracts in mg/mL.

	PGSEA	PGSP	PGFPE	PGLEA	PGLPE	PGSM	PGLM	DTH2O	DTM	DTEA	DTPE	ALM	ALEA	ALP	ArM	ArEA	ArPE	DMSO	GENT
MIC	0.391	1.563	6.250	0.391	3.125	0.781	1.563	6.250	3.125	1.563	12.50	3.125	0.391	3.125	0.390	0.390	1.563	25.00	0.098
MBC	1.563	12.500	25.000	3.125	12.50	3.125	6.250	25.00	12.50	6.250	25.00	12.50	3.125	12.50	3.125	1.563	12.50	>50.00	0.195

PGSEA = *P. granatum* seed ethyl acetate, PGSP = *P. granatum* seed pet ether, PGFPE = *P. granatum* fruit peel ethyl acetate, PGLEA = *P. granatum* leaf ethyl acetate, PGLPE = *P. granatum* leaf pet ether, PGSM = *P. granatum* seed methanol, PGLM = *P. granatum* leaf methanol, DTH_2_O = *D. kilimandscharicus* tuber water, DTM = *D. kilimandscharicus* tuber methanol. DTEA = *D. kilimandscharicus* tuber ethyl acetate, DTPE = *D. kilimandscharicus* tuber pet ether, ALM = *A. secundiflora* leaf methanol, ALEA = *A. secundiflora* leaf ethyl acetate, ALP = *A. secundiflora* leaf pet ether, ArM = *A. rabaiensis* leaf methanol, ArEA = *A. rabaiensis* leaf ethyl acetate, ArPE = *A. rabaiensis* leaf pet ether, DMSO = Dimethyl sulphoxide, GENT = Gentadox

**Table 2:** Results of brine shrimps lethality test against plant extracts.

Sample code	LC_50_ (mg/mL)	95% CI (mg/mL) Lower limit–Upper limit.	Regression equation	Regression coefficient (*R*^2^)
DTW	3.519 × 10^−3^	(2.363–5.241) × 10^−3^	*Y* = 49.69logx + 22.845	0.9541
ArW	5.016 × 10^−1^	(3.259–7.713) × 10^−1^	*Y* = 63.25logx−120.8	0.9984
ASM	3.902 × 10^−1^	(2.678–5.684) × 10^−1^	*Y* = 74.388logx−142.76	00.9915
DTM	7.242 × 10^−2^	(5.969–8.785) × 10^−2^	*Y* = 112.24logx−158.75	0.9462
PGFPEA	1.720 × 10^−1^	(1.418–2.086) × 10^−1^	*Y* = 145.2logx−274.6	0.9954
PGFPM	1.245	(6.46.8505 × 10^−1^)-2.396	*Y* = 42.763logx−82.357	0.983
ALPE	1.896 × 10^−2^	(1.451–2.477) × 10^−2^	*Y* = 81.124logx−53.664	0.9744
PGLM	1.512 × 10^−1^	(1.203–1.90.129) × 10^−1^	*Y* = 105.93logx−180.89	0.8841
DTPE	7.937 × 10^−4^	(6.147 × 10^−4^)–(1.2911 × 10^−3^)	*Y* = 49.834logx + 55	1.0
ALEA	7.645 × 10^−3^	(5.5784 × 10^−3^)-(1.048 × 10^−2^)	*Y* = 58.151logx−1.3704	0.9129
PGSM	2.082 × 10^−1^	(5.779 × 10^−2^)–(7.498 × 10^−1^)	*Y* = 80.141logx−135.8	0.9033
PGFPE	5.505 × 10^−2^	(4.691–6.460) × 10^−2^	*Y* = 151.57logx−213.85	0.9808
PGLEA	1.245	(6.469 × 10^−1^)-2.396	*Y* = 42.763logx−82.357	0.983
PGLPE	2.302 × 10^−1^	(1.634–3.243) × 10^−1^	*Y* = 70.705logx−117.01	0.8536
DTEA	1.468 × 10^−2^	(1.079–1.998) × 10^−2^	*Y* = 64.321logx−25.053	0.9899
PGSEA	6.428 × 10^−3^	(4.4896–9.2022) × 10^−3^	*Y* = 55.168logx + 5.421	0.9485
CLPM	1.637 × 10^−2^	(1.201–2.231) × 10^−2^	*Y* = 69.9680logx−34.9360	0.994929

DTEA = *D. kilimandscharicus* tuber ethyl acetate, DTPE = *D. kilimandscharicus* tuber pet ether, ALM = *A. secundiflora* leaf methanol, ALEA = *A. secundiflora* leaf ethyl acetate, ALP = *A. secundiflora* leaf pet ether, ArW = *A. rabaiensis* leaf water, PGSEA = *P. granatum* seed ethyl acetate, PGFPE = *P. granatum* fruit peel ethyl acetate, PGLEA = *P. granatum* leaf ethyl acetate, PGLPE = *P. granatum* leaf pet ether, PGSM = *P. granatum* seed methanol, PGLM = *P. granatum* leaf methanol, DTH_2_O = *D. kilimandscharicus* tuber water, DTM = *D. kilimandscharicus* tuber methanol, CLPM = Cyclophosphamide.

**Table 3:** Percentage of chickens with Salmonella spp. identified by microscopical and biochemical tests from both wards.

Microscopically		Biochemical test		
*Salmonella* isolates	Non-*Salmonella* isolates	*S. gallinarum*	Upland Tengeru	Lowland Tengeru
162/360 (45%)	198/360 (55%)	138/360 (38.3%)	60/360 (16.7%)	78/360 (21.6%)

**Table 4:** Biochemical reactions of *Salmonella* spp.

Test	Results	
	Upland Tengeru	Lowland Tengeru
Motility	Nonmotile	Nonmotile
Gram staining	Positive	Positive
Colon arrangement	Singly or pair	Singly or pair
Urease	Negative	Negative
Catalase	Positive	Positive
Oxidase	Negative	Negative
McConkey agar	Pale yellow transparent colony	Pale yellow transparent colony
TSI	Alkaline slant, acid butt	Alkaline slant, acid butt
H_2_S production	Positive	Positive

*Sugar utilization*		
Maltose	Positive	Positive
Dulcitol	Positive	Positive
Fructose	Positive	NT
Sucrose	Negative	Negative
Lactose	Negative	Negative
Dextrose	Positive	Positive
Glucose	Positive	Positive
Mannitol	Positive	Positive
Biotype	*S. gallinarum*	*S. gallinarum*

NT-Not tested.

## Data Availability

The data are available from the corresponding author upon request.
